# Gradient Mn-La-Pt Catalysts with Three-layered Structure for Li-O_2_ battery

**DOI:** 10.1038/srep34950

**Published:** 2016-10-12

**Authors:** Kedi Cai, Rui Yang, Xiaoshi Lang, Qingguo Zhang, Zhenhua Wang, Tieshi He

**Affiliations:** 1 Liaoning Engineering Technology Research Center of Supercapacitor, Bohai University, Jinzhou 121013, China; 2 School of Chemical Engineering and Environment, Beijing Institute of Technology, Beijing 100081, China; 3 School of Materials Science & Engineering, Georgia Institute of Technology, Atlanta, GA 30332-0295, USA

## Abstract

Gradient Mn-La-Pt catalysts with three-layered structure of manganese dioxide (MnO_2_), lanthanum oxide (La_2_O_3_), and Platinum (Pt) for Li-O_2_ battery are prepared in this study. The mass ratio of the catalysts is respectively 5:2:3, 4:2:4, and 3:2:5 (MnO_2_: La_2_O_3_: Pt) which is start from the side of the electrolyte. The relationship between morphology structure and electrochemical performance of gradient catalyst is investigated by energy dispersive spectrometry and constant current charge/discharge test. The Li-O_2_ battery based on gradient Mn-La-Pt catalysts shows high discharge specific capacity (2707 mAh g^−1^), specific energy density (8400 Wh kg^−1^) and long cycle life (56 cycles). The improvement of the Li-O_2_ battery discharge capacity is attributed to the gradient distribution of MnO_2_ and Pt and the involvement of La_2_O_3_ that can improve the energy density of the battery. More important, this work will also provide new ideas and methods for the research of other metal-air battery.

Li-O_2_ battery is a kind of metal–air battery that employs lithium as the anode electrode and oxygen in the air as the cathode electrode reactant. The Li-O_2_ battery has received worldwide attention because of its higher theoretical specific capacity for a rechargeable battery^1^,^2^,^3^,^4^,^5^. The advantage of the Li-O_2_ battery with existing battery technology is the higher energy density of lithium metal (3860 mAh g^−1^), as a result of its electrochemical reaction with oxygen in the air^6^,^7^,^8^. The Li-O_2_ battery is consisted of three main parts which are lithium metal anode, electrolyte and porous carbon cathode.

In fact, the actual specific energy of the Li-O_2_ battery, which is far from the theoretical value, is restricted by many factors^9^,^10^,^11^. The cathode air electrode can greatly affect the many performance issues of entire battery, therefore, the study of the cycle ability and stability of Li-O_2_ battery is mainly focused on the research of cathode catalysts^12^,^13^,^14^,^15^,^16^. To find an efficient cathode catalyst that can enhance the activity of oxygen reduction reaction (ORR) and oxygen evolution reaction (OER) is essential^17^,^18^,^19^,^20^,^21^. In the Li-O_2_ battery, the porous cathode electrode is utilized to store the solid products formed by the reactions of lithium ion and O_2_ during the discharge^22^,^23^,^24^,^25^,^26^. The catalyst can open the solid reactant, thereby promoting the discharge and cycle of the battery. Metal oxide catalyst plays a major role in discharge of battery, a noble metal catalyst at the time of charging shows good electrochemical activity^27^ and lanthanide catalyst can increase the voltage plateau of battery.

Up to now, a lot of work is focused on cathode structure and electrolyte material for Li-O_2_ battery. The catalyst is the key component of the battery, which have not been studied in detail. We proposed the gradient catalysts with three-layered structure of manganese dioxide (MnO_2_), lanthanum oxide (La_2_O_3_), and Platinum (Pt) for Li-O_2_ battery in this work. The influences of the novel catalyst on the performance of battery were also investigated. The surface morphologies and structure of the electrodes were observed by scanning electron microscopy (SEM) and energy dispersive spectrometry (EDS). The mass transfer resistances and the charge and discharge performances of the battery were characterized by electrochemical impedance spectra (EIS) and constant current charge/discharge tests, respectively.

## Results and Discussion

### The characterization of catalysts

**Figure 1:**
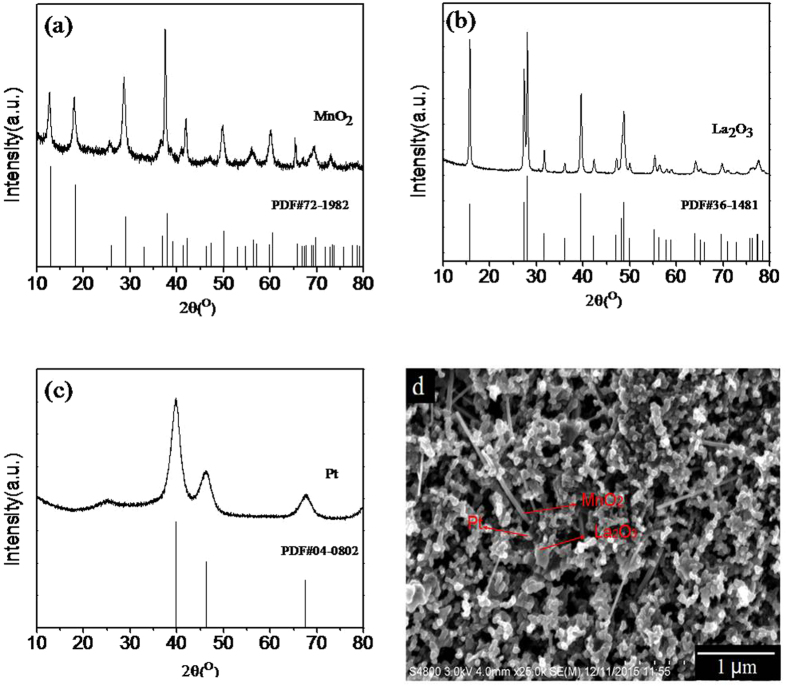
XRD patterns of catalysts: (**a**) MnO_2_, (**b**) La_2_O_3_, (**c**) Pt/C and (**d**) SEM image of the catalysts.

Figure 1a–c show the XRD patterns of the three catalysts respectively. Compared with the standard cards, the diffraction peaks of the samples are in completely agreement with the characteristic peak of the standard card, and three kinds of homemade catalysts are determined as manganese dioxide, lanthanum oxide and carbon supported platinum. The SEM image of the cathode electrode of the Li-O_2_ battery with the gradient catalyst is shown in Fig. 1d. Figure 1d shows that the three catalysts are uniformly distributed to surface of this carbon paper. The rod-like structure is considered manganese dioxide (MnO_2_), the scale-like structure is regarded as lanthanum oxide (La_2_O_3_) and the granular structure is carbon supported platinum (Pt/C). This condition is in favor of ORR and OER.

**Figure 2:**
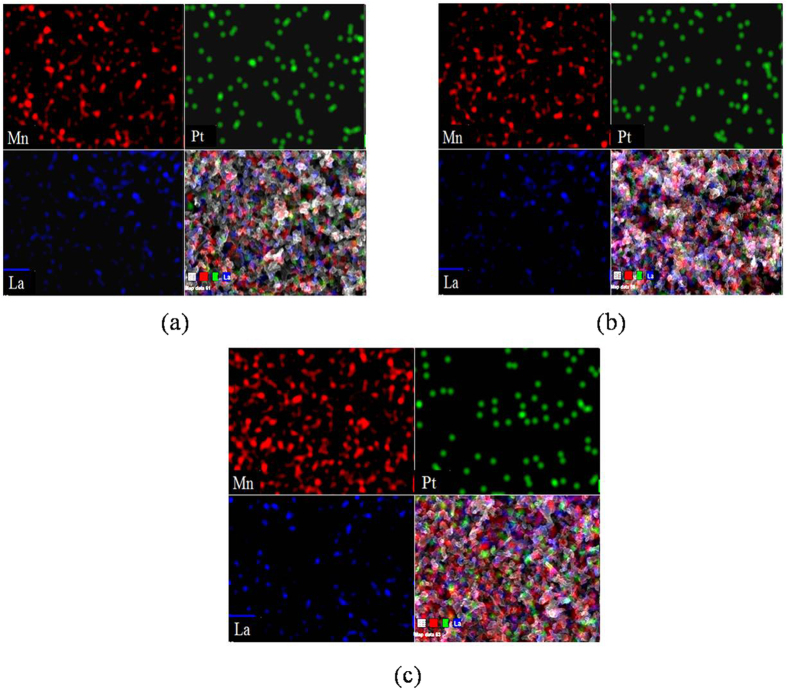
EDS surface scanning images of first-layer catalyst layer (**a**), second-layer catalyst layer (**b**) and third-layer catalyst layer (**c**).

Figure 2 shows the energy dispersive spectrometry (EDS) images of the gradient cathode material. A comprehensive analysis of the samples is carried out here. The three spectrograms of sample content represent morphology of the first-layer, the second-layer and the third-layer catalyst layer, respectively. It can be seen from the three figures that the content of Mn is increased, but the content of Pt is reduced. In Fig. 2b, the La content is much less than the other two components of Mn and Pt. It indicates that the content of the three elements of each layer is approximately the same as the content of the prepared. So, the sprayed slurry is relatively uniform on the carbon paper.

**Figure 3:**
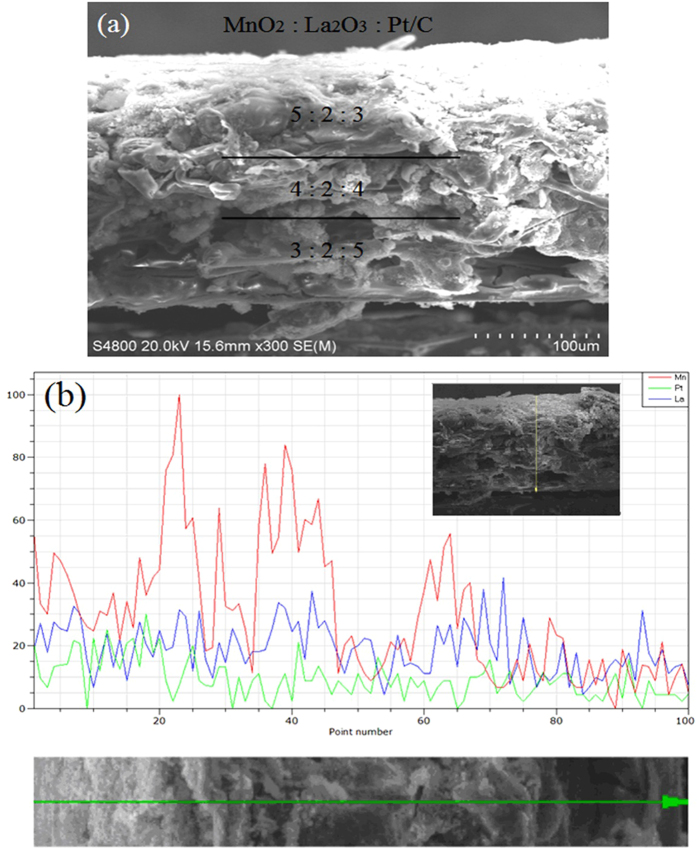
Sectional scanning images of SEM (**a**) and EDS (**b**).

The sectional scanning images of SEM are shown in Fig. 3. It can be clearly seen that the three layer structures of the cathode from the Fig. 3a. Figure 3b shows the peak intensities of the Mn, La and Pt three elements for the underlined part from left to right. The red line, green line and blue line represent Mn, Pt and La respectively which indicates the trend of peak intensities for three elements. Mn shows a downward trend in the whole trend of Mn and La appears trend of lower in both sides and higher in the middle, but there is no change in the trend of Pt. In the first-layer catalyst layer, peak intensity of Pt is reduced, and there is a possible reason that Pt is actually Pt/C and is close to the carbon paper. Owing to there is much interspace among the carbon paper. Pt is likely to enter the interspace of carbon paper, so the peak intensity of Pt is relatively low.

### Electrochemical test of gradient Mn-La-Pt catalysts

**Figure 4:**
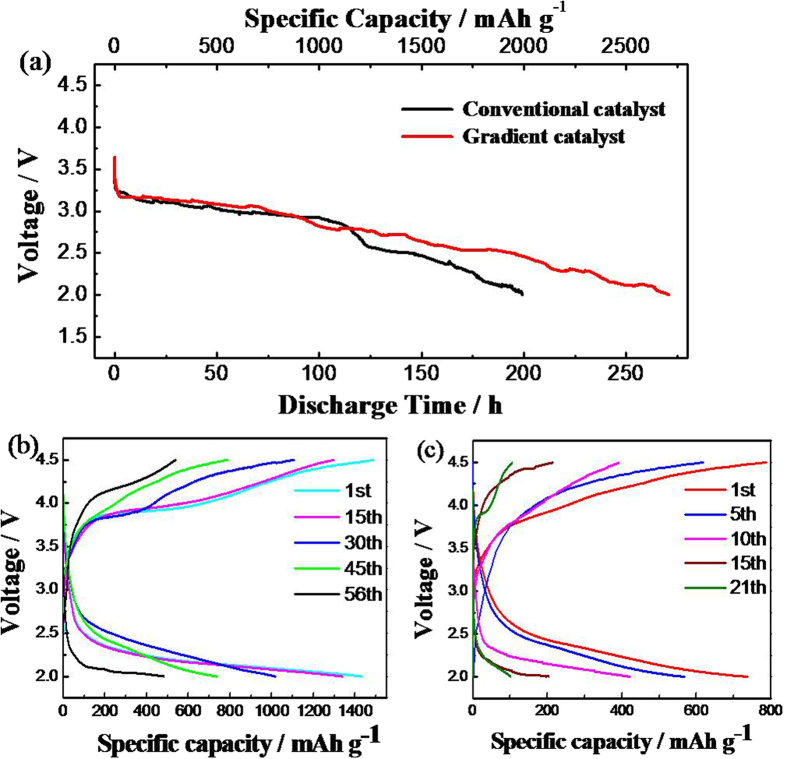
Initial discharging curves of Li-O_2_ batteries with gradient and conventional catalysts at 0.01 mA cm-2 and 25 °C (**a**), constant current charge/discharge curves of Li-O_2_ batteries with gradient catalyst (**b**) and constant current charge/discharge curves of Li-O_2_ batteries with conventional catalyst (**c**) at different cycle times.

It can be seen the initial discharging curves of the Li-O_2_ batteries with gradient and conventional catalysts at 0.01 mA cm^−2^ and 25 °C from the Fig. 4a. The initial discharge time of the Li-O_2_ battery with the gradient Mn-La-Pt catalysts is 270.7 h (2707 mAh g^−1^) higher than 200.5 h (2005 mAh g^−1^) of the conventional catalyst. The constant current charge/discharge curves of the batteries with gradient and conventional catalysts are shown in Fig. 4b,c at different cycle times. Long time work can be up to 56 cycles for the battery with gradient catalyst. Compared with traditional Li-O_2_ battery, the Li-O_2_ battery with a gradient catalyst exhibits a relatively excellent discharge performance because the gradient Mn-La-Pt catalyst is applied in the design of the Li-O_2_ battery. The mass ratio of the catalysts is 5:2:3 (MnO_2_: La_2_O_3_: Pt), 4:2:4, and 3:2:5, which is start from the side of the electrolyte. In the gradient catalyst structure, MnO_2_ plays an important role in the discharge process and Pt/C has great effects on the charging process. The MnO_2_ shows good catalysis property for ORR, and Pt is a good catalyst for OER. La_2_O_3_ can improve the discharge plateau. In the discharge and charging processes, the Li-O_2_ battery using a gradient catalyst can effectively provide a special site for electrochemical reaction. MnO_2_, La_2_O_3_, and Pt/C distribute orderly on the catalyst layer of the cathode that is advantageous to cycle performance and long-time operation of Li-O_2_ battery.

**Figure 5:**
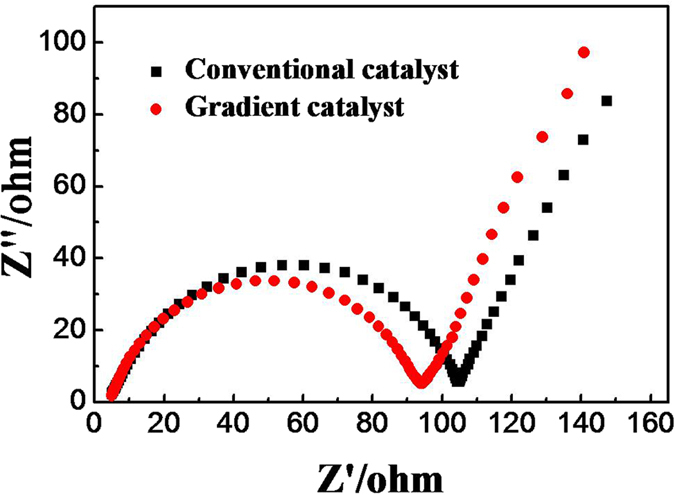
EIS of the gradient and traditional catalysts at a cell voltage of 2.4 V and temperature of 25 °C.

AC impedance test is performed on the gradient catalyst and conventional catalyst at the voltage of 2.4 V and 25 °C. Nyquist diagrams of the batteries can be seen from the Fig. 5, the high-frequency region is a half arc and the low-frequency region is almost a straight line. The internal resistance of the battery can be obtained from the intersection of high frequency on the real axis, and the mass transfer resistance of the batteries can be obtained from the low frequency region of the Nyquist diagram, with the increase of the high frequency radius, and the resistance of mass transfer increases. Figure 5 shows that the mass transfer resistance of porous cathode is lower than that of traditional cathode. The Li-O_2_ battery using the gradient catalyst is beneficial to the electrochemical reaction and the mass transfer of oxygen.

### Power and energy density test of gradient Mn-La-Pt catalysts

**Figure 6:**
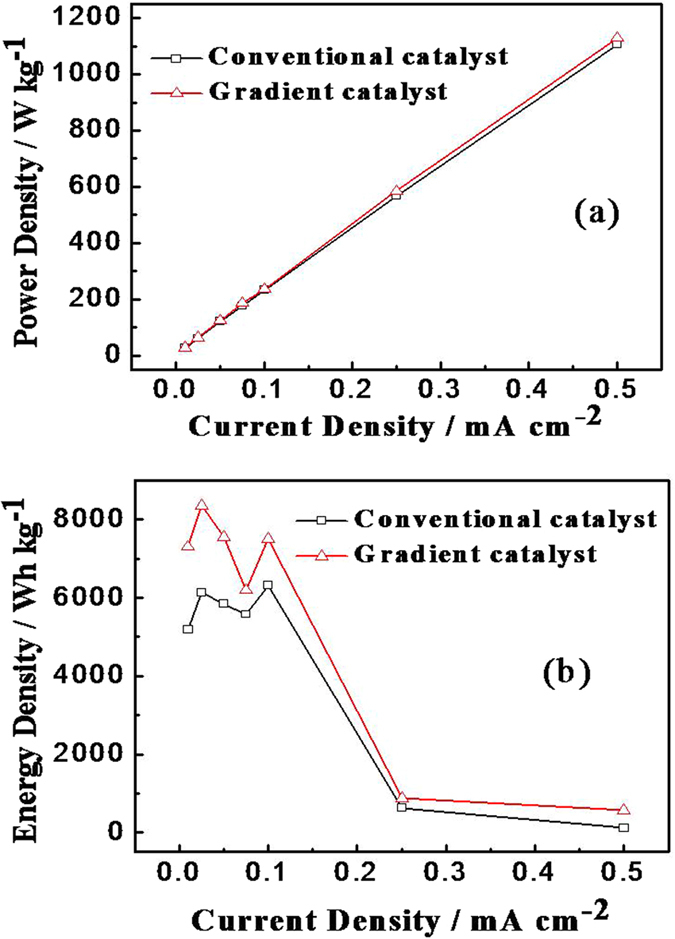
(**a**) Power density curves of Li-O_2_ batteries with gradient and conventional catalysts at 25 °C and (**b**) Energy density curves of Li-O_2_ batteries with gradient and conventional catalysts at 25 °C.

Figure 6a,b show the power and energy density curves of the Li-O_2_ batteries with gradient catalyst and conventional catalyst at different current densities at 25 °C. As shown in the Fig. 6a, the power density increases with the increase of the current density, and the power density of the gradient catalyst is slightly higher than that of the traditional catalyst. These results show that the discharge platform of assembled battery is stable and the structure of the cathode has a little influence on the Li-O_2_ battery power density. Figure 6b shows the curve graph of the energy density changes with the current density in the batteries. The two curves are undulating at 0.01–0.1 mA cm^−2^ and the curves decrease with current density at 0.1–0.5 mA cm^−2^. The phenomenon is attributed to the high severity of electrode polarization at a high current density. Results show that the energy density of Li-O_2_ battery with a three-layered composite gradient catalyst as the cathode was 8400 Wh kg^−1^, and the battery with a conventional catalyst is 6300 Wh kg^−1^. La_2_O_3_ can improve the discharge plateau. The gradient catalyst exhibits better specific energy than the conventional catalyst at different current densities that due to the involvement of La_2_O_3_ improve the energy density of the battery.

## Conclusions

A three-layered composite gradient catalyst as a cathode catalyst for a Li-O_2_ battery was successfully synthesized in this study. It allows the large capacity of the rechargeable Li-O_2_ battery and is high to 2707 mAh g^−1^ at the initial discharge. The improvement of the Li-O_2_ battery discharge capacity is attributed to the gradient distribution of MnO_2_ and Pt and the involvement of La_2_O_3_ can improve the energy density of the battery. Long-term work can be up to 56 cycles. Compared with the traditional Li-O_2_ battery, this battery showed higher capacity and recharge ability. MnO_2_, La_2_O_3_, and Pt/C distribute orderly on the catalyst layer of the cathode and can be utilized to improve discharge capacity effectively and extend the cycle life of Li-O_2_ battery.

## Experimental Section

### Preparation of catalysts and cathode

- MnO_2_ was prepared through the following process: 2.5 g of MnSO4·H2O (Sinopharm Chemical Reagent Co., Ltd.) and 1.5 g of KMnO4 (Sinopharm Chemical Reagent Co., Ltd.) were added to 20 mL deionized water to fully dissolve. Firstly, 100 mL deionized water was added to a three-neck flask, and the oil bath was heated to 95 °C. Secondly, the two kinds of solutions were slowly added to the three-neck flask from the two sides and stirred constantly. After adding the two kinds of solution, both sides of the flask were blocked with a ground glass stopper, heated to 95 °C, and continued for 18 h under this temperature until a brown suspension was obtained. Thirdly, the suspension was cooled at room temperature, and static precipitation was allowed to occur for 1 h. The samples were oscillated for 10 min under ultrasonic wave to make the precipitation disperse evenly and for filtration to occur. Water was then added, and several times of dispersion and filtration were implemented. Finally, the resulting catalyst was dried for 12 h at 80 °C in a vacuum. The MnO_2_ catalyst was obtained.
- The detailed La_2_O_3_ preparation process was as follows. A total of 2.6 g of lanthanum nitrate (Sinopharm Chemical Reagent Co., Ltd.) and 1.4 g of citric acid (Sinopharm Chemical Reagent Co., Ltd.) were dissolved in 5 mL deionized water. The two kinds of solutions were added to a third beaker slowly at the same time. Then, the 5 mL deionized water was added. Until the pH of the mixed solution was 2.0, the mixed solution was placed in a furnace. The resulting catalyst was calcinated for 1.5 h at 700 °C in a muffle furnace. The La_2_O_3_ catalyst was then obtained.
- The Pt/C catalyst was prepared by impregnation reduction method. Here, the catalyst precursor was hexachloroplatinic acid (H_2_PtCl_6_) (Shanghai Aladdin Reagent Co., Ltd.) and reducing agent was formaldehyde (Shanghai Aladdin Reagent Co., Ltd.). The detailed steps of the preparation process were as follows^28^: firstly, the 30 ml of water and 30 ml of isopropanol (Shanghai Aladdin Reagent Co., Ltd.) were added to 50 mg Vulcan XC-72 (Shanghai King Chemical Co., Ltd.), and the suspension was ultrasonically stirred for 1 h. Then, add H_2_PtCl_6_ to the suspension and stir ultrasonically for 2 h, the pH value of the suspension was adjusted to 12 by NaOH aqueous solution, then adding excessive formaldehyde to the suspension drop by drop. The mixture was sonicated for 20 min and then heated to 80 °C, it was agitated continuously for 3 h under this temperature. At last, the prepared catalyst was washed by ultra pure water until the chloride ion was not detected. The Pt/C catalyst was dried at 110 °C for 24 h in a vacuum drying oven. The Pt/C catalyst of 40 wt. % was obtained.

The oxygen electrode consists of a gas diffusion layer and three layer catalysts in this article. The preparation process of gas diffusion layer was as follows: The carbon paper (Shanghai River Electric Co., Ltd.) was soaked in Polytetrafluoroethylene emulsion (PTFE) (5 wt. %, DuPont Corporation) for 20 min and allowed to dry naturally. Afterward, carbon paper was dried at 350 °C. The preparation of the catalyst layer was as follows: The three kinds of the homemade catalysts, the super P conductive black and PTFE binder were made into the uniformly slurry and then spray to the gas diffusion layer, and the mass ratio of Super P/catalysts/binder was 70/20/10. The all operation steps were carried out at 100 °C and with the 2 atm. The prepared oxygen electrode was placed in a vacuum drying oven for 24 h. The carbon loading of oxygen electrode was 1.0 mg cm^−2^.

The catalyst ratio of the three layer catalysts is different during the preparation of the electrode. The mass ratio of the catalyst near the electrolyte side was 5:2:3 (MnO_2_: La_2_O_3_: Pt/C), that of the catalyst near the cathode side was 3:2:5, and between the two layers was 4:2:4. The ratio of the carbon loading of the three layer catalysts was 2:1:2.

### Fabrication of battery with gradient Mn-La-Pt catalysts

Lithium sheet (16 mm diameter, 0.2 mm thick) was used as anode electrode of Li-O_2_ battery (Tianjin Lithium Industry Co., Ltd.). The oxygen electrode was cut into a circle with a diameter of 10 mm as cathode electrode. The electrolyte (2 mol L^−1^) was consisted of commercial electrolyte and lithium tetrafluoroborate (the molar ratio of 1:3). The polypropylene microporous membrane was cut into circles with a diameter of 16 mm as separators. The conventional catalyst that mentioned in the text was composed of MnO_2_ and Pt/C.

**Figure 7:**
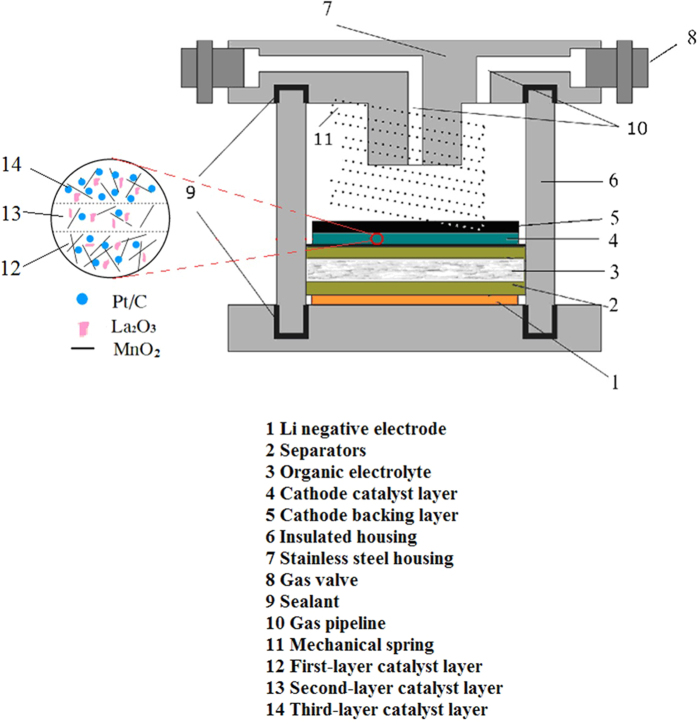
Structure diagram of the Li-O_2_ battery.

The structure of the battery was shown in Fig. 7. The assembly process of the battery was carried out in a glove box filled with argon atmosphere. The stainless steel on the bottom of the fixture was just as the current collector of the negative electrode, the foam nickel was used as the current collector of the positive electrode. The main function of the diaphragms is to isolate the positive and negative electrode. The effect of the stainless steel spring is to ensure better contact between the internal and external circuits. The battery was allowed to stand for 1 h before testing.

### Physical characterization of catalysts and cathode

Scanning electron microscopy (SEM) is used to scan the sample surface by clustered electron beam, which uses the interaction between the electron and the sample, collects the secondary electrons and the scattered electron from the surface of the sample, and converts it to the electrical signal, finally displays the image that is synchronized with the emission electron beam. The physical and chemical information can be directly observed on the surface of the sample by SEM, such as morphology, composition, crystal structure and so on. The SEM (Hitachi S-4800) was used to observe the surface morphology of the electrode in this paper. Energy dispersive spectrometry (EDS) can be analyzed for the types and content of elements, because each element has a specific X-ray characteristic wavelength, which is determined by the characteristic energy released during the energy level transition.

X-ray powder diffraction (XRD) is a kind of research method, which is through X-ray diffraction of materials, analysis of the diffraction pattern, and to obtain information about the composition of the materials, the structure or form of internal atoms or molecules. The used instrument was Rigaku Ultima IV (Japan) in this paper.

### Electrochemical measurements of Li-O_2_ battery

AC impedance test of the Li-O_2_ battery was measured at the voltage of 2.4 V for 25 °C by electrochemical analyzer (CHI 660E) which frequency range from 100 kHz to 0.1 Hz. alternating voltage amplitude of AC impedance test was 5 mV. It can be roughly divided into high frequency and low frequency regions.

The Li-O_2_ battery was tested in the voltage range of 2 V to 4.5 V by the NEWARE cycler (Shenzhen Neware Electronic Co. Ltd.) at different current densities. All processes of the assembly battery were carried on a glove box with dry and pure oxygen at the constant current density of 0.01 mA cm^−2^ (0.025, 0.05, 0.075, 0.1, 0.25, and 0.5 mA cm^−2^).

## Additional Information

**How to cite this article**: Cai, K. *et al*. Gradient Mn-La-Pt Catalysts with Three-layered Structure for Li-O_2_ battery. *Sci. Rep.* **6**, 34950; doi: 10.1038/srep34950 (2016).
